# Supplementary material for: Rationale and study protocol of a regional health panel in Saxony, Germany (GEPASA)
Source: PLoS One. 2024 Nov 15;19(11):e0310656. doi: 10.1371/journal.pone.0310656 (PMC11567545; doi:10.1371/journal.pone.0310656)
Supplement: S2 File — (PDF) [file pone.0310656.s002.pdf]

## Perceived quality of life in a region

1. **In general terms: how satisfied are you with the city or community in which you live?**

| dissatisfied | | | | | | satisfied |
|---|---|---|---|---|---|---|
| 1 | 2 | 3 | 4 | 5 | 6 | 7 |

| Anticipated use | Source |
|---|---|
| global query of perceived quality of life in a region and dependent variable for the study of the influence of perceived quality of care on perceived quality of life in a region | population survey by the Federal Institute for Re-search on Building, Urban Affairs and Spatial Devel-opment in Germany 2012 |

## Perceived health status

**2. How satisfied are you with your health?**

| comple-tely dis-satisfied | | | | | | | | | comple-tely sa-tisfied |
|---|---|---|---|---|---|---|---|---|---|
| 1 | 2 | 3 | 4 | 5 | 6 | 7 | 8 | 9 | 10 |

| Anticipated use | Source |
|---|---|
| global query of satisfaction with health (not time-bound) to be able to re-late satisfaction to perceptions of quality of care | German Socio-Economic Panel (SOEP) 2020 |

**3. How would you describe your health in general?**

- very good
- good
- satisfactory
- poor
- very poor

| Anticipated use | Source |
|---|---|
| global assessment of health status as a predictor of perceived quality of life in a region, perceived quality of health care, and utilization of health care services | German General Social Survey (ALLBUS) 2014 and 2018 |

**4. To what extent are you permanently limited because of an illness in performing your daily tasks?**

- not limited
- limited, but not severely
- severely limited
- I don’t know.

| Anticipated use | Source |
|---|---|
| GALI question (Global Activity Limitation Indicator) as a predictor of perceived quality of life in a region, perceived quality of health care, and utilization of health care services | • Health Study on German adults (DEGS) 2009<br>• European Union Statistics on Income and Living Conditions (EU-SILC) 2019 |

**5. In the last month, have you been sad or depressed?**
yes/no

| Anticipated use | Source |
|---|---|
| question concerning depression as a predictor of perceived quality of life in a region, perceived quality of health care, and utilization of health care services | Survey of Health, Ageing and Retirement in Europe (SHARE) 2004 |

**6. Have you felt too feeble to do things you would have liked to do in the last month?**
yes/no

| Anticipated use | Source |
|---|---|
| question concerning depression as a predictor of perceived quality of life in a region, perceived quality of health care, and utilization of health care services | Survey of Health, Ageing and Retirement in Europe (SHARE) 2004 |

## Health problems

7. **Here we have a list of common illnesses.**
   **Please tell me which diseases you have been suffering from for at least 12 months or chronically (i.e. even longer).**

   - high blood pressure, hypertension
   - heart diseases (e.g. angina pectoris, heart attack, arrhytmias)
   - liver diseases (e.g. inflammation of the liver, hepatitis, liver shrinkage, liver cirrhosis)
   - kidney diseases (e.g. renal insufficiency)
   - gastrointestinal diseases (e.g. chronic gastritis, irritable bowel syndrome)
   - neurological diseases (e.g. Parkinson's disease, multiple sclerosis)
   - dementia, Alzheimer’s disease
   - stroke
   - chronic illness of the eye (e.g. cataract, myopia, hyperopia)
   - chronic illness of the ear (e.g. hearing loss)
   - diabetes
   - cancer
   - allergy
   - food intolerance
   - atopic dermatitis
   - migraine
   - chronic pain (other than migraine)
   - rheumatism, chronic joint inflammation, arthritis, arthrosis, gout
   - damage to the spine
   - osteoporosis
   - chronic bronchitis
   - asthma
   - depression
   - anxiety disorder
   - jaw and dental diseases
   - other disease

   - no answer

| Anticipated use | Source |
|---|---|
| diagnosed diseases, in order to be able to explain utilization of health care services and perception of quality of care and to differentiate between per-ceived health problems and diagnosed diseases | German General Social Survey (ALLBUS) 2014 |

8. **Please select each of the health problems from the list that you have had in the last 12 months, even if they have been present for some time. These do not need to be diagnosed.**

Please also tell us in each case to what extent you are or were permanently limited by the condition in carrying out your daily tasks (professional and private).

| | did not have this in the last 12 months | had this in the last 12 months | did not limit me | did limit me, but not severely | did limit me severely |
|---|---|---|---|---|---|
| heart or circulation problems (e.g. high blood pressure, arrhythmias) | | | | | |
| urological problems (e.g. urinary tract infections, kidney stones) | | | | | |
| stomach or digestive problems | | | | | |
| problems with vision (e.g. due to age) | | | | | |
| problems with hearing (e.g. due to age) | | | | | |
| breathing problems such as asthma attacks, wheezing or whistling breathing | | | | | |
| skin problems (e.g. itching, dry skin or acne) | | | | | |
| migraine or severe headache | | | | | |
| back or neck pain | | | | | |
| muscular or joint pain | | | | | |
| infectious disease (such as flu) | | | | | |
| corona infection | | | | | |
| prolonged symptoms 3 months after corona infection | | | | | |
| injuries and/or fractures | | | | | |
| sleeping problems (such as difficulty falling asleep or restless sleep) | | | | | |
| forgetfulness | | | | | |
| mental stress (e.g. anxiety, dejection) | | | | | |
| gynecological problems (e.g. menstrual cramps) | | | | | |
| dental problems | | | | | |
| allergies | | | | | |

| | | | | | |
|---|---|---|---|---|---|
| addiction problems (also gambling addiction) | | | | | |

| Anticipated use | Source |
|---|---|
| symptoms and symptom severity to be able to explain utilization and perception of quality of care | • European Social Survey (ESS) 2014<br>• Health Study on German adults (DEGS) 2009<br>• German General Social Survey (ALLBUS) 2014 |

## Utilization of health care services

**9. Do you have a general practitioner who you see first for health problems?**

- yes
- no
- no answer

| Anticipated use | Source |
|---|---|
| crucial to measure extent of utilization of medical services; general practitioner as gatekeeper in the German healthcare system | Health Study on German adults (DEGS) 2009 |

**10. For what reason or reasons have you seen a doctor in the past three months?**
You can select several occasions.

- because of an acute illness (e.g. flu or injury)
- because of a chronic inllness (e.g. diabetes, high blood pressure/hypertension, rheumatism)
- because I felt unwell (e.g. general discomfort, sleep disorders)
- for requesting advice
- for a visit to the doctor's practice, but without consulting the doctor (e.g. because I needed to get a prescription, radiotherapy)
- due to anxiety, dejection and/or irritability
- for a preventive medical check-up or screening (e.g. skin cancer screening)
- for a vaccination

| Anticipated use | Source |
|---|---|
| crucial to capture extent of utilization of medical services | German General Social Survey (ALLBUS) 2014 |

**11. Which doctors did you see for one of the reasons just mentioned (question 10)? You can select more than one doctor.**

- general practitioner
- surgeon
- orthopedist
- gynecologist
- dermatologist
- ENT physician
- neurologist
- urologist
- ophthalmologist
- anesthesist (in the context of pain therapy)
- internist (e.g. cardiologist, pulmonologist, nephrologist, gastroenterologist)
- psychiatrist
- psychologist
- company physician

| Anticipated use | Source |
|---|---|
| crucial to capture extent of utilization of medical services | • European Social Survey (ESS) 2014<br>• list of doctors taken from European Health Interview Survey (EHIS) 2010 |

12. **Have you seen any of the following therapists in the last 12 months?**

    Please tick each therapist on the list below to whom this applies.

    - physiotherapist
    - occupational therapist
    - speech therapist
    - psychotherapist
    - dietician
    - podiatrist
    - alternative practitioner
    - diabetes consultant

| Anticipated use | Source |
|---|---|
| crucial to capture extent of utilization of medical services | European Health Interview Survey (EHIS) 2010 |

13. **Have you visited a hospital emergency room within the last 12 months?**

    yes/no

14. **If so, please also tell us why.**
    You can select multiple answers.

    - It was a health emergency.
    - I did not get an appointment with any general practitioner or specialist.
    - I was not admitted as a new patient to any general practitioner.
    - The doctor was on vacation or sick.
    - I did not know that there was an on-call doctor's practice.
    - I was not sure which practice to go to.
    - The on-call doctor's practice was too far away.
    - no answer

| Anticipated use | Source |
|---|---|
| crucial to measure extent of use of medical services and to relate this to perceived quality of health care at place of residence | German General Social Survey (ALLBUS) 2014 |

15. **For how many nights were you admitted to a hospital for inpatient treatment in the last 12 months?**

    - about _______ nights
    - none at all

| Anticipated use | Source |
| --- | --- |
| crucial to measure extent of use of medical services and to relate this to perceived quality of health care at place of residence | Health Study on German adults (DEGS) 2009 |

## Perceived quality of health care

**16. In general, how would you rate the quality of medical care where you live?**

| | …excellent | …very good | …good | …suffi-cient | …poor |
|---|---|---|---|---|---|
| I find the quality of medical care where I live is... | | | | | |

| Anticipated use | Source |
|---|---|
| global query of the perceived quality of care at the place of residence | International Health Policy Survey (IHP-Survey) Swit-zerland 2020 |

**17. In the last 12 months, were you ever unable to get a medical treatment you needed for any of the reasons on the list below?**

Please indicate each reason on the list below that applies to you. You may also choose more than one reason.

- other private or occupational commitments
- way to the needed treatment too far
- fear of medical examinations and treatments
- no appointments or treatment options available
- no suitable doctor found
- doctor's practice not available for appointments (neither by phone, e-mail or in person)
- wrong referral or medical information
- no interest in long waiting times in the waiting room
- no referral to the specialist from my general practitioner
- I have gotten every appointment with a doctor and every treatment I have needed.
- I have not needed any appointment with a doctor and any treatment in the last year.
- I wanted to wait to see if the symptoms would get better on their own.
- no answer

| Anticipated use | Source |
|---|---|
| • predictors of perceived quality of care in the outpatient sector: ap-pointment availability and availability of treatment places<br>• Personal predictors (items 1, 3, and 10) of medical service utilization as interference variables for explaining perceived quality of care and utili-zation behavior<br>• fallback categories | • European Social Survey (ESS) 2014<br>• European Union Statis-tics on Income and Liv-ing Conditions (EU-SILC) 2019 |

**18. Where you live, how easy or difficult is it to get medical care for acute medical problems in the evening, on weekends, or on holidays without going to a hospital emergency room?**

| | ...very easy. | ...quite easy. | ...quite difficult. | ...very difficult. | I have never needed care in the evening, week-ends or holidays. | I don't know. | no ans-wer |
|---|---|---|---|---|---|---|---|
| Based on my experi-ence, where I live, this is... | | | | | | | |

| Anticipated use | Source |
|---|---|
| predictor of perceived quality of care in the outpatient sector: availability of treatment slots on non-working days for acute complaints | International Health Policy Survey (IHP-Survey) Swit-zerland 2020 |

**19. Now we would like to know how long it takes you to get from your home to one of the following doctors and therapists if you use your usual means of transport (e.g. car, public transport, bicy-cle)?**

You do not have to be in treatment there.

| | Time in minutes | |
|---|---|---|
| general practitioner | | I don't know. |
| surgeon | | |
| orthopedist | | |
| gynecologist | | |
| dermatologist | | |
| ENT physician | | |
| neurologist | | |
| psychotherapist | | |
| urologist | | |
| ophthalmologist | | |
| anesthesist (in the context of pain ther-apy) | | |
| psychiatrist | | |
| internist (e.g. cardiologist, pulmonolo-gist, nephrologist, gastroenterologist) | | |
| physiotherapist | | |

| | | |
|---|---|---|
| occupational therapist | | |
| speech therapist | | |
| dietician | | |
| podiatrist | | |
| pharmacy | | |

| Anticipated use | Source |
|---|---|
| predictor of perceived quality of care in the outpatient sector: distance to medical service provision | • Healthcare-Seeking in Germany (HEALSEE) 2014<br>• list of doctors taken from European Health Interview Survey (EHIS) 2010 |

**20. When you last visited a practice, how long did you have to wait for an appointment? This is <u>not</u> about an emergency.**

| | up to 24 hours | up to 1 week | up to 1 month | up to 3 mon-ths | more than 3 months | more than 6 mon-ths | I never got an ap-point-ment. | I did not need an ap-point-ment. | I have used the emer-gency ser-vice of the Asso-cia-tion of Statu-tory Health Insur-ance Physi-cians. |
|---|---|---|---|---|---|---|---|---|---|
| primary practicioner | | | | | | | | | |
| surgeon | | | | | | | | | |
| orthopedist | | | | | | | | | |
| gynecologist | | | | | | | | | |
| dermatologist | | | | | | | | | |

| | | | | | | | | | |
|---|---|---|---|---|---|---|---|---|---|
| ENT doctor | | | | | | | | | |
| neurologist | | | | | | | | | |
| psychotherapist | | | | | | | | | |
| urologist | | | | | | | | | |
| ophthalmologist | | | | | | | | | |
| anesthesist (in the context of pain therapy) | | | | | | | | | |
| psychiatrist | | | | | | | | | |
| internist (e.g. cardiologist, pulmonologist, nephrologist, gastroenterologist) | | | | | | | | | |
| physiotherapist | | | | | | | | | |
| occupational therapist | | | | | | | | | |
| speech therapist | | | | | | | | | |
| dietician | | | | | | | | | |
| podiatrist | | | | | | | | | |

| Anticipated use | Source |
|---|---|
| predictor of perceived quality of care in the outpatient sector: availability of appointments for non-acute complaints | • International Health Policy Survey (IHP-Survey) Switzerland 2020<br>• European Social Survey (ESS) 2014 |

**21. In the last 12 months, has it happened to you that...**

| | yes | no | I don't know. | no answer |
|---|---|---|---|---|
| ... you have received contradictory information from different physicians or medical staff? | | | | |
| ... you have felt that a medical test ordered by the doctors is superfluous because it has already been done? | | | | |

| Anticipated use | Source |
|---|---|
| irregularities during medical consultations as a predictor of quality of care | International Health Policy Survey (IHP-Survey) Switzerland 2020 |

**22. In the last 12 months, was your last visit to a health care provider a visit to…**

- a general practitioner?
- a medical specialist?

**23. Please think about this last visit to a doctor. Then please tell us to what extent you agree with the following statements.**

| | fully agree | rather agree | rather do not agree | do not agree at all | I don't know. | was not relevant |
|---|---|---|---|---|---|---|
| She or he had important information about my medical history (e.g. my medical record). | | | | | | |
| She or he has spent enough time with me. | | | | | | |
| She or he involved me in decisions about my care and treatment to the extent I wished her or him to. | | | | | | |
| She or he explained things to me in a way that was easy to understand. | | | | | | |

| Anticipated use | Source |
|---|---|
| predictors of perceived quality of care in the outpatient sector: shared decision making and information gaps | International Health Policy Survey (IHP-Survey) Switzerland 2020 |

**24. Now we are talking about the costs of medicines, aids or individual health services that were offered to you by a doctor in the last 12 months.**
Please select each of the following statements that apply to you.

I have…

- ... not sought an individual health service (e.g. a vaccination before a trip or measurement of intraocular pressure) due to the costs involved.
- ... not sought dental treatment or a dental examination because of the costs involved.
- ... not picked up a prescribed medication due to the costs involved.
- … not used a prescribed remedy, e.g. for physiotherapy, because of the costs involved.
- ... not redeemed a prescribed aid, e.g. glasses or a hearing aid, because of the costs involved.

- I have not been offered any medications, aids, or individual health services in the past 12 months.

| Anticipated use | Source |
|---|---|
| perceived deprivation as an explanatory variable to assess the perceived quality of care | Survey of Health, Ageing and Retirement in Europe (SHARE) 2004 |

25. **Now, we would like to know if you have needed a scheduled surgery within the last 24 months.**
    Scheduled surgeries are those that are not due to an emergency. For example, a scheduled surgery would be a knee or shoulder joint procedure (for an arthroplasty or a joint replacement).
    It does not matter whether this scheduled surgery was performed in a hospital (inpatient) or in a doctor's office (outpatient).

- yes
- no

- no answer

| Anticipated use | Source |
|---|---|
| crucial to capture extent of utilization of medical services | International Health Policy Survey (IHP-Survey) Switzerland 2020 |

26. **ONLY IF QUESTION 24 WAS ANSWERED WITH YES: How long did you have to wait for your scheduled surgery appointment?**

- less than a week
- more than a week but less than a month
- more than a month but less than three months
- more than three months but less than a year
- one year or longer

- I don’t know.
- no answer

| Anticipated use | Source |
|---|---|
| predictor of perceived quality of care in the outpatient and inpatient sector: availability of appointments for scheduled surgeries | International Health Policy Survey (IHP-Survey) Switzerland 2020 |

## Everyday behavior

27. **What means of transport do you normally use in everyday life?**
    You can state more than one means of transport.

    - public transport (e.g. bus or streetcar)
    - car
    - motorcycle, moped, scooter
    - bicycle
    - on foot
    - carpooling

    - no answer

| Anticipated use | Source |
|---|---|
| everyday mobility to assess the perceived quality of care and to map every-day behavior | German Socio-Economic Panel (SOEP) 2020 |

28. **On how many of the last 7 days did you either walk quickly or otherwise engage in physical activity for 30 minutes or more?**
    For instance, this could have been sports, gardening, or a physically demanding job.

    on ___ of the last 7 days

| Anticipated use | Source |
|---|---|
| physical activity as a dimension of everyday behavior | European Social Survey (ESS) 2014 |

29. **How many hours do you sleep on a regular day?**
    Please also include naps during the day.

    On a usual day I sleep ___ hours.

| Anticipated use | Source |
|---|---|
| sleep behavior as a dimension of everyday behavior | German Socio-Economic Panel (SOEP) 2020 |

30. **And now it is about smoking cigarettes. Which of the following descriptions best describes your smoking behavior?**

    - I smoke daily.
    - I smoke occasionally, but not daily.
    - I do not smoke anymore, but I used to smoke.
    - I have smoked only a few times.
    - I have never smoked.

    - I have been or I am exposed to secondhand smoke several times a week (because someone around me smokes).

    - No answer

| Anticipated use | Source |
|---|---|
| smoking behavior as a dimension of everyday behavior | European Social Survey (ESS) 2014 |

**31. How often do you consume the following foods?**
The list includes both food and beverages.

| | more than once a day | once a day | more than once a week | at least once a week | at least once a month | less than once a month | never | I can-not eat this due to an al-lergy or an in-toler-ance. |
|---|---|---|---|---|---|---|---|---|
| potatoes, pasta, rice | | | | | | | | |
| bread or rolls | | | | | | | | |
| vegetables or salad (excluding potatoes) | | | | | | | | |
| fruit and/or berries (excluding fruit juices and smoothies) | | | | | | | | |
| white meat (chicken, turkey or goose) | | | | | | | | |
| red meat (pork, beef or game) | | | | | | | | |
| sausage products | | | | | | | | |
| fish and other sea-food | | | | | | | | |
| eggs | | | | | | | | |
| dairy products such as cheese, curd, yo-gurt or butter | | | | | | | | |
| convenience foods (e.g. instant noodles and frozen pizza) | | | | | | | | |
| sweets and/or sweet pastries (e.g. cakes and pies) | | | | | | | | |
| beer (including shandy) and/or wine | | | | | | | | |
| liquor and/or spirits (e.g. rum, whiskey or cocktails) | | | | | | | | |
| beverages containing caffeine and tea (e.g. coffee, black tea or mate) | | | | | | | | |
| soft drinks (e.g. Coke® or Fanta®) | | | | | | | | |

| | | | | | | | | |
|---|---|---|---|---|---|---|---|---|
| energy drinks (e.g. Red Bull®) | | | | | | | | |
| juices or juice spritz-ers and smoothies | | | | | | | | |
| water | | | | | | | | |

| Anticipated use | Source |
|---|---|
| dietary behavior as a dimension of everyday behavior | • European Social Survey (ESS) 2014<br>• German General Social Survey (ALLBUS) 2014 |

**32. Does one of these statements apply to you?**

- I eat exclusively vegetarian.
- I eat exclusively vegan.

| Anticipated use | Source |
|---|---|
| dietary behavior as a dimension of everyday behavior | self-developed |

**33. How often have you taken the following medications in the last three months?**
In addition, for each medication, please tell us whether it was prescribed to you by a doctor or not.

| | have not taken this | have taken this less than once a month | have taken this less than once a week | have taken this once a week | have taken this more than once a week | have taken this daily | was pre-scribed to me by a doctor | was **not** pre-scribed to me by a doctor |
|---|---|---|---|---|---|---|---|---|
| painkillers (e.g. As-pirin®, Paraceta-mol®, Voltaren® or Ibuprofen®) | | | | | | | | |
| sleeping pills (e.g. zopiclone, zolpidem) | | | | | | | | |
| sedatives (e.g. Tavor ®, diazepam) | | | | | | | | |

| Anticipated use | Source |
|---|---|
| use of prescription drugs as a dimension of everyday behavior | Epidemiological Survey of Substance Abuse (ESA) 2012-2021 |

## Insurance status

**34. What is your health insurance status?**

- statutory health insurance (without private supplementary insurance)
- statutory health insurance with private supplementary insurance
- exclusively private health insurance
- private health insurance with allowance
- no health insurance

- no answer

| Anticipated use | Source |
|---|---|
| insurance status as a predictor variable to classify perceived quality of care. | self-developed |

## Body measurements

**35. How tall are you without shoes?**
Please state your height in meters and centimeters.

___ meters and ___ centimeters

| Anticipated use | Source |
|---|---|
| body mass index as a predictor of perceived health status, perceived quality of life, and perceived burden of disease | German General Social Survey (ALLBUS) 2014 |

**36. What is your current weight without clothes?**

___ kg

| Anticipated use | Source |
|---|---|
| body mass index as a predictor of perceived health status, perceived quality of life, and perceived burden of disease | German General Social Survey (ALLBUS) 2014 |

## Education

**37. What is your highest school degree?**

- lower secondary school/elementary school certificate/POS class 8/9
- secondary school leaving certificate/POS class 10
- higschool degree/EOS
- I do not have a school degree.
- No answer

| Anticipated use | Source |
|---|---|
| level of education as a dimension of living conditions | German General Social Survey (ALLBUS) 2014 |

**38. Which professional or academic training did you complete?**
If you do not find yourself in the selection below, please select the degree that most closely matches yours.

- completion of recognized professional training
- master craftsman/technician or comparable technical college degree
- bachelor's degree
- master's degree, Diplom, Magister, state examination
- I do not have a vocational or academic training qualification.
- No answer

| Anticipated use | Source |
|---|---|
| level of education and training as a dimension of living conditions | occupational code of the German statuatory health insurance 2010, positions 6 and 7 |

## Occupation

**39. Now it is about your professional activity. Which of the activities from the list below best describes yours?**

I am…

- …full-time employed.
- …half-time employed.
- …part-time employed.
- …school student.
- ...university student.
- ...pensioner / retiree.
- ...housewife / househusband.
- ...currently unemployed.
- ...in voluntary military service / federal voluntary service / FSJ / FÖJ.
- ...not (full-time) employed for other reasons

| Anticipated use | Source |
|---|---|
| occupation as a predictor variable to classify everyday behavior, perceived health status, and utilization | German General Social Survey (ALLBUS) 2018 |

**40. Please tell us how much you are exposed to the following strains at work.**
Please think only of your current or last professional activity.
If you have never had a job, you can skip this and the next question.
*Attending school or studying does not qualify as "professional activities".*

| | none | little | moderate | high | does not apply to my work |
|---|---|---|---|---|---|
| Stress due to working at night and in shifts | | | | | |
| physical strain (e.g. due to heavy lifting) | | | | | |
| psychological strain (e.g. due to stress, being overworked or mobbing) | | | | | |
| stress due to an insecure employment situation or existential fears | | | | | |
| stress due to noise | | | | | |
| stress due to a long commute | | | | | |

| Anticipated use | Source |
|---|---|
| proxy for stress due to occupation and thus a predictor variable to classify everyday behavior, perceived health status, and utilization | self-developed based on the Study on Mental Health at Work (S-MGA) 2011/12 |

**41. Please tell us to what extent you have freedom of choice in your work.**

*Explanation: Activities with no freedom of decision are relatively simple activities for which no lengthy training or learning period is required and which always follow the same pattern, e.g. assembly line work.*

*Activities with a high degree of freedom of decision are usually more complex. For instance, this often applies to self-employed activities.*

| | no… | little… | moderate… | high… |
|---|---|---|---|---|
| …freedom of choice | | | | |

| Anticipated use | Source |
|---|---|
| proxy for psychological stress due to occupational activity and thus a predictor variable to classify everyday behavior, perceived health status, and utilization | self-developed based on the Job Content Questionnaire (JCQ) 1998 |

## Household

**42. How many people live in your household, including yourself?**
This means all persons who live permanently in your household and who either depend on or contribute to the household's income.

___ persons | no answer

| Anticipated use | Source |
|---|---|
| basis for calculating deprivation | German National Cohort (NAKO) 2014 |

**43. How many of them are 14 years old or younger?**

___ persons | no answer

| Anticipated use | Source |
|---|---|
| basis for calculating deprivation | German National Cohort (NAKO) 2014 |

**44. How many people under 18 (children) live with you in the alternating model?**
"Alternating model" means that the persons do not live with you every day, but live in another household on some days or in some weeks.

___ persons | no answer

| Anticipated use | Source |
|---|---|
| patchwork households as a basis for calculating deprivation | self-developed |

**45. What is the total monthly net income of your household?**
This is the sum that remains after deduction of taxes and social security contributions.
Please also include all so-called transfer payments, for example unemployment benefits, social benefits, housing allowances and alimony (e.g. child support).

___ €

If you cannot or do not want to state the exact amount, please place yourself in one of the categories below.

- under 500 € ......................................................................
- 500 € to under 750 € ......................................................
- 750 € to under 1.000 € ....................................................
- 1.000 € to under 1.250 € .................................................
- 1.250 € to under 1.500 € .................................................
- 1.500 € to under 1.750 € .................................................
- 1.750 € to under 2.000 € .................................................
- 2.000 € to under 2.250 € ................................................
- 2.250 € to under 2.500 € ................................................
- 2.500 € to under 3.000 € ................................................

- 3.000 € to under 3.500 € ................................................
- 3.500 € to under 4.000 € ................................................
- 4.000 € to under 4.500 € ................................................
- 4.500 € to under 5.000 € ................................................
- 5.000 € to under 6.000 € ................................................
- 6,000 € to under 8,000 € ................................................
- over 8,000 € ....................................................................
- no answer

| Anticipated use | Source |
|---|---|
| basis for calculating deprivation | German General Social Survey (ALLBUS) 2014 |

## Relationship status

**46. Now we have one question about your relationship status. Are you...**

- … single?
- … in a life partnership, with your partner living in the same household?
- … in a life relationship, with your partner living in a different household?

- no answer

| Anticipated use | Source |
|---|---|
| possible predictor for health care utilization | German General Social Survey (ALLBUS) 2018 |

## Gender

**47. Which gender do you identify yourself with?**

- female
- male
- diverse

- no answer

| Anticipated use | Source |
|---|---|
| possible predictor for health care utilization | German General Social Survey (ALLBUS) 2018 |

## Age

**48. What year were you born?**

_______

| Anticipated use | Source |
|---|---|
| possible predictor for health care utilization | German General Social Survey (ALLBUS) 2014 |
