# Supplementary material for: Rationale and study protocol of a regional health panel in Saxony, Germany (GEPASA)
Source: PLoS One. 2024 Nov 15;19(11):e0310656. doi: 10.1371/journal.pone.0310656 (PMC11567545; doi:10.1371/journal.pone.0310656)
Supplement: Attachment — Submitted filename: P2P reply_fin.docx [file pone.0310656.s003.docx]

| No | Comment | Reply |
| --- | --- | --- |
|  | Reviewer #1 | |
| 1 | This manuscript is titled as "Rationale and Study Protocol ..." and provides a lot of information about the background, preliminary work (e.g. questionnaire design), hypotheses, and aspects of design and analysis. However, in my view some of this information is too extensive or does not necessarily belong here while other is missing. However, the planned panel study may yield very interesting and relevant results and the authors are encouraged to provide a revised version which is somewhat more focussed. | Thank you for recognising the relevance and potential impact of our panel. We are also grateful for the opportunity to resubmit our paper to PLOS ONE.  We fully understand your concerns about the length of our paper and have therefore significantly shortened the manuscript.  As for the information that was missing from our original manuscript, we have included it in the resubmitted version. In the following, we describe the changes we have made to the initial submission based on the valuable input from you and your reviewer. |
| 2 | The first part consisting of Introduction/Rationale/Objective/Hypotheses is with 10 pages excessively long. In addition, it would help if the central constructs health status and quality of life were more clearly defined and distinguished, e.g. do you mean health-related QoL or QoL more generally?  In this part, nine objectives are stated and later on eleven hypotheses. Some of these (e.g. H5 to H8) are not very original and have been covered by many other studies, others are more pertinent. Some refer to "the regional level", others not, and it is not always clear which ones refer to a cross-sectional or longitudinal perspective. I will not go into more detail but challenge the authors to structure this part in a better way while concentrating on the major study questions | As stated above, we fully understand that the detailed process of how the hypotheses were derived is too lengthy for a study protocol. We appreciate your suggestion to shorten this part and such focused on key research questions. Given the fact that our research is mainly descriptive and such non-confirmatory, we decided to concentrate on our main target, addressing changes in central constructs over time and aiming to describe the reality of health care delivery in Saxony from a citizens’ perspective, rather than going into details on potential hypotheses. The central research questions are justified within the section “rationale” of our manuscript (p. 6f) and read as follows:  *“The overarching research questions of the panel are:*   1. *What is the relationship between perceptions of health status, quality of health care and quality of life?* 2. *Do individual (health) behavior, medical diagnoses, use of health care facilities, work environment and socio-demographic factors influence perceptions of health status, quality of health care and quality of life?* 3. *How do perceptions of health status, quality of health care and quality of life change over time?”*   As for the central constructs of our research, we have now listed the central domains within the questionnaire, complete with definitions and the suggested relationships between them:   - *“Perceived overall quality of life in the region of residence (dependent variable used for studying the influence of perceived quality of health care on quality of life at the region of residence)* - *Perceived health status and health problems (perceived health problems and specific diagnoses; predictor for utilization of health care services and perceived quality of health care)* - *Utilization of health care services (predictor for perceived quality of health care)* - *Perceived quality of health care (predictor for quality of life at the region of residence and central construct of interest of the panel survey)* - *Behavioral habits (predictor for perceived health status and utilization of health care services)* - *Education, occupation and household status (dimension of living conditions; predictor for utilization of health care services and perceived health status)* - *Demographics (insurance and relationship status, gender, age, weight, height) (predictor for utilization of health care services and perceived health status)”* |
| 3 | Also, an improved version of Fig.1 might help where constructs relating to the individual and those relating to the region are distinguished. | Thank you for this valuable suggestion, which we have applied to figure 1. The figure now shows the interrelationships addressed by our research questions and covers the potential levels of analysis (individual, within region and interregional), as well as changes over time. |
| 4 | In the methodology part of the paper, I was missing the age range for participants. | Thank you for pointing this out. As we have added in the methodology part, we are recruiting participants aged 18 and above, with no upper threshold. |
| 5 | The statistical analysis plan is very vague and does not mention the very important aspect of how to deal with drop-out. | Thank you for raising the issue of drop-outs. To address this issue, we are aiming to recruit n = 3,000 participants, which results in an oversampling of 33% compared to the calculated sample size of n = 2,250. |
| 6 | The authors state that Bonferroni-Holm method is used to adjust for error inflation due to multiple testing, but with the large number of heterogenous hypotheses it remains unclear how this will be done (how many hypothesis tests, is there a hierarchy...). | Thank you for this important comment, which we fully acknowledge. However, as we have now refrained from formulating complex hypotheses and instead focused on three central research questions, as you kindly suggested, a more concrete description of the statistical plan is not feasible at this time. We therefore list a number of potentially relevant statistical analyses with reference to our research questions (see section “Statistical Plan” on p. 14). |
| 7 | The sample size calculation is a bit unspecific and would be more relevant if there were a clear primary hypothesis. While a response rate of 25% is ambitious but can be achieved with intensive recruitment efforts, a drop-out rate of only 25% after 6 years seems very optimistic. | Thank you for encouraging us to clarify our sample size calculation. As you pointed out, sample size calculations are usually based on a primary hypothesis. However, as we still needed a recruitment target despite our exploratory approach, we have now clearly indicated that our sample size calculation is based on an exemplary change in a binary construct – hence the logistic regression as an exemplary statistical analysis. We have also lowered our expected response rate to 15% based on pre-testing. We aim to address attrition by oversampling for the first wave of the panel, as described above (no. 5). |
| 8 | Questionnaire construction and pretesting is described over 5 pages, although pretesting is not finished yet. In my view, the information in the current manuscript could be shortened and an extensive description of item selection, pretesting and piloting would make an interesting separate paper. | Thank you for suggesting a separate paper describing the development of the questionnaire. We followed your suggestion and have removed all information on this topic, including pretesting, from the manuscript. |
| 9 | Do the authors have empirical data showing that the average time for answering the questionnaire is only 30 mins? The questionnaire may be divided in 48 questions, but these include so many subquestions that the final number of items exceeds 180! I would assume that a pilot test of the questionnaire will result in a much more practical version. | We appreciate your detailed feedback on our project. Indeed, the questionnaire is quite extensive, however, all questions are multiple or single choice item scales and such quick to fathom and quickly answered. In fact, the pre-test questionnaire took no more than 30 minutes to complete. Nevertheless, adaptions to the questionnaire were made after the pretest. |
| 10 | Table 1 is not self-explanatory and should be better described. What does Sample = 312.5 mean? | Thank you for pointing out the shortcomings of this important table. We have now clarified the columns more detailed in the text, which reads as follows:  *“The final sample consists of 48 clusters. For each of the 48 clusters formed, 312 to 313 persons are randomly selected for initial contact. For this purpose, a random sample will be requested from the residents' registration offices for each district or municipality.”* |
| 11 | There are some typos, e.g. line 72 "renumeration" and line 94 "setteling". | Thank you for reading our manuscript with such diligence. We have corrected the typos. |
|  | Reviewer #2 | |
| 12 | In your abstract, you write ‘We aim to conduct a longitudinal panel study of the perceived health status and perceived quality of health care of the Saxon citizens as a basis for the systematic derivation of health care goals/measures and target group-specific, regionally suitable prevention measures.’ I can very well understand that a survey can help to derive such goals and or measures. However, I do not see that the survey that you propose can serve this purpose. To design a survey that actually serves this purpose, one would have to start with an idea as to which kind of information could be applied in which way to derive the aforementioned goals and/or measures. You do not present such an idea. Instead, you present in the sub-chapter ‘Objectives’ research questions that hardly relate to the purpose formulated in the abstract and that, in themselves, do not constitute a systematic research conception. | Thank you for taking the time to review our project so thoroughly. We agree that, deriving strategic goals for health care planning is a complex and presumably multi-methodological process and can therefore only be a subsequent step to the analysis of the data we aim to gain through our panel. Therefore, in both the abstract and the objectives of our paper, we simply state that the panel data can be used as a basis for such a process.  In terms of the research questions that guide our work, we have formulated three central questions (as shown above) which represent the scope of our research. All other – and more detailed – research questions have been removed from the manuscript.  Strategic goals for health planning are now only mentioned in the Background and Discussion section. |
| 13 | The relationship between the questions and figure 1 remains unclear and figure 1 in itself does not convey a clear idea of the research objectives. | Thank you for pointing out some shortcomings of our central figure. We have redesigned Figure 1 so that it now includes our three central research questions, their assumed interrelationships and the regional as well as spatial dimensions within our work. For more details see no. 3. |
| 14 | I also see no reason why you formulate hypotheses in this context. For the planning of measures it is in no way important whether the empirical results accord to a-priori formulated hypotheses. | Thank you for encouraging us to refrain from detailed hypotheses. Instead, we clarify that our work is mainly exploratory, focusing on three key research questions, as outlined above. |
| 15 | You determine the sample size for logistic regression without clarifying which role logistic regression plays in this context. Some chapters later, you even say that you will use ANOVAs for your main research question. This means that you determine your samples size for a statistical methods that does not seem to be very important in the intended research and that you intend to apply, for the questions you seem to see as most important, a statistical method for which you have not determined the sample size. | Thank you for giving us the opportunity to explain our sample size calculation in more detail. We see your points about the statistical analysis which we have chosen as the target method of calculation and the variable that it concerns, we now make it very clear that both are exemplary. This is because we are following an exploratory research design, but still need a target for recruitment. However, as regression analyses will be fundamental for our work, we chose this procedure as the exemplary one for our sample size calculation. We have therefore moved the section on sample size planning further to the top of the section on statistical analysis in our paper. |
| 16 | Moreover, you plan to combine the survey with qualitative research. In principle, this is a fine idea. However, you make in no way clear as to how you will relate the results of the survey to the results of the qualitative research. | Thank you again for reading our manuscript so carefully and for raising this important point. As stated above (no. 12), we no longer refer to the development of strategic health care goals. Consequently, qualitative methods have been removed from the manuscript. |
